# Effect of Mesopore Development on Butane Working Capacity of Biomass-Derived Activated Carbon for Automobile Canister

**DOI:** 10.3390/nano11030673

**Published:** 2021-03-09

**Authors:** Byeong-Hoon Lee, Hye-Min Lee, Dong Chul Chung, Byung-Joo Kim

**Affiliations:** 1 Research Center for Environmental Materials, Korea Institute of Carbon Convergence Technology, Jeonju 54853, Korea; byeonghoon1004@naver.com (B.-H.L.); hmlee2014@hanmail.net (H.-M.L.); 2 Department of Organic Materials & Fiber Engineering, Jeonbuk National University, Jeonju 54896, Korea; 3 Department of Nano & Advanced Materials Engineering, Jeonju University, Jeonju 55069, Korea

**Keywords:** kenaf, activated carbon, phosphoric acid, butane working capacity

## Abstract

Kenaf-derived activated carbons (AKC) were prepared by H_3_PO_4_ activation for automobile canisters. The microstructural properties of AKC were observed using Raman spectra and X-ray diffraction. The textural properties were studied using N_2_/77 K adsorption isotherms. Butane working capacity was determined according to the ASTM D5228. From the results, the specific surface area and total pore volume of the AKC was determined to be 1260–1810 m^2^/g and 0.68–2.77 cm^3^/g, respectively. As the activation time increased, the butane activity and retentivity of the AKC increased, and were observed to be from 32.34 to 58.81% and from 3.55 to 10.12%, respectively. The mesopore ratio of activated carbon increased with increasing activation time and was observed up to 78% at 973 K. This indicates that butane activity and retentivity could be a function not only of the specific surface area or total pore volume, but also of the mesopore volume fraction in the range of 2.8–3.8 nm and 5.5-6.5 nm of adsorbents, respectively. The AKC exhibit enhanced butane working capacity compared to commercial activated carbon with the high performance of butane working capacity due to its pore structure having a high mesopore ratio.

## 1. Introduction

For the past several decades, fossil fuels have been used as the main energy source, but this has caused serious air pollution [1,2]. Renewable energy has been studied to replace fossil fuels, and bioenergy (bio-hydrogen [3], bio-gas [4], bio-ethanol [5], and biodiesel [6]) is receiving much attention in particular. Bioenergy using biomass produced by absorbing and fixing carbon dioxide in the air through photosynthesis is expected to replace existing oil or gas [7,8,9]. Among various biomasses, kenaf is a very useful precursor with a high yield per unit area, fast growth rate, and high CO_2_ adsorption [8,9]. Kenaf can be classified into seeds, leaves, and stems. Seeds and leaves are used for high added value, such as biodiesel [9] and medicine [10], respectively, while stems are mainly used as fuel pellets [11] or livestock feed [12]. Therefore, many studies have attempted to increase the value of kenaf stems to biochar or activated carbon [13,14]. Although research [15,16,17] is underway to manufacture activated carbon derived from kenaf, only research results on low-quality activated carbon with low yield and low pore characteristics due to high holocellulose content ratio have been published. In many studies, most of the kenaf-derived activated carbons have a specific surface area of less than 500 m^2^/g. Activated carbon with a specific surface area of less than 500 m^2^/g is closer to biochar than activated carbon. Activated carbon with a specific surface area of less than 500 m^2^/g has very insufficient adsorption capacities to be applied to environmental or energy applications. Therefore, it is necessary to study high quality activated carbon derived from kenaf with excellent pore characteristics. In order to develop kenaf-based activated carbon with excellent pore characteristics, it is necessary to examine other activation methods in addition to the existing activation methods focusing on the oxidation of crystals.

Recently, environmental regulations are increasingly being strengthened, and in order to meet this, activated carbon having better pore characteristics is required [18]. Activated carbon has been applied to the evaporative emission control system (EVAP) canister for the reduction of unburned hydrocarbons (HC) [19]. To meet the California’s Low Emission Vehicle Program (LEV-3), introduced in 2018, activated carbon with better pore characteristics is required [18]. The evaporated gas is mainly generated in the fuel tank of the vehicle when parking or refueling the vehicle [20]. The evaporated gas is adsorbed on the activated carbon of the canister, and when driving, the adsorbed gas is purged from activated carbon and burned in the engine [19]. Therefore, the activated carbon of the canister that repeats adsorption and desorption requires excellent adsorption and desorption capacity and is known to depend on the micropore volume and the mesoporosity, respectively [21].

In this study, kenaf-derived activated carbons for automobile evaporative system with excellent pore characteristics was prepared by using phosphoric acid activation. Stabilization of phosphate before activation was used for high yield and excellent textural properties of kenaf-derived activated carbons (AKC). The effect of phosphoric acid activation conditions on the pore characteristics was researched by observing microstructure and pore structure analysis. In addition, the pore structure that determines the performance of the carbon canister was confirmed through the correlation analysis between the textural properties and butane working capacity (BWC).

## 2. Experimental

### 2.1. Sample Preparation

The kenaf used in this study was provided by the Jeonbuk-do Agricultural Research & Extension Service. The kenaf was crushed after drying to less than 2% water content and sieved to a particle size fraction of 1–2 cm. The kenaf was immersed in phosphoric acid (H_3_PO_4_, 85%, Daejung Chemicals & Metals CO., Siheung, Korea) and stabilized to stand at 298–473 K for 24 h for impregnation treatment. The stabilized kenaf was heated to the activation temperature under a nitrogen atmosphere (200 mL/min) in a tubular furnace. The reactor temperature was increased at a rate of 10 K/min until reaching the activation temperature (673–1073 K). After reaching the activation temperature, the stabilized kenaf was activated for 60 min, and it was allowed to cool naturally. After the sample was cooled to below 373 K, the activated kenaf was washed in boiling distilled water to remove the phosphoric acid. The activated kenaf was named AKC–activation temperature to show the experimental conditions. This label shows that the activated kenaf-derived activated carbons (AKC) were manufactured at activation temperature.

### 2.2. Characterizations

Thermogravimetric analysis was performed using a (TGA-50, SHIMADZU, Tokyo, Japan) with an N_2_ flow at a heating rate of 10 K/min up to 1273 K. The Microstructure of AKC was determined using a wide-angle X-ray diffractometer (XRD, X’Pert^3^ Powder, Malvern Panalytical, Almelo, The Netherlands) with Cu Kα (1.542 A) source over a 10–60° range at a rate of 2°/min. The crystallite size of the AKC was calculated using the Scherrer equation [22].

*L* = Kλ/*B*cos*θ* (1)

The textural properties of AKC were observed using isotherm adsorption/desorption curves of N_2_/77 K (BELSORP-max, BEL JAPAN, Osaka, Japan). Before the observation, AKC was put into a cylinder cell and degassed overnight while maintaining a residual pressure below 10^−3^ bar at 573 K. The specific surface area (S_BET_) was calculated from the isothermal adsorption curves based on the Brunauer–Emmett–Teller (BET) equation [23]. Pore size distribution was calculated using the non-localized density functional theory (NLDFT) [24].

The butane working capacity (BWC) of the AKC was measured according to ASTM-D5228 standard [25]. After 16.7 mL of AKC was filled in a U-shaped tube, the mass difference according to the saturation and purgation of butane was measured in a constant temperature water bath at 298 K. The mass difference between the saturated and purged AKC is the BWC in weight of butane per weight of AKC. BWC, butane activity, and butane retentivity were calculated using Equations (2)–(4).

(2) $$\mathrm{Butane}\;\mathrm{working}\;\mathrm{capacity}\;(\%)=\frac{\mathrm{weight}\;\mathrm{of}\;\mathrm{butane}\;\mathrm{adsorbed}\;\mathrm{AC}-\mathrm{weight}\;\mathrm{of}\;\mathrm{the}\;\mathrm{nitrogen}\;\mathrm{purged}\;\mathrm{AC}}{\mathrm{weight}\;\mathrm{of}\;\mathrm{the}\;\mathrm{AC}}\times 100$$

(3) $$\mathrm{Butane}\;\mathrm{activity}\;(\%)=\frac{\mathrm{weight}\;\mathrm{of}\;\mathrm{butane}\;\mathrm{adsorbed}\;\mathrm{AC}-\mathrm{weight}\;\mathrm{of}\;\mathrm{the}\;\mathrm{AC}}{\mathrm{weight}\;\mathrm{of}\;\mathrm{the}\;\mathrm{AC}}\times 100$$

(4) $$\mathrm{Butane}\;\mathrm{retentivity}\;(\%)=\frac{\mathrm{weight}\;\mathrm{of}\;\mathrm{the}\;\mathrm{nitrogen}\;\mathrm{purged}\;\mathrm{AC}-\mathrm{weight}\;\mathrm{of}\;\mathrm{the}\;\mathrm{AC}}{\mathrm{weight}\;\mathrm{of}\;\mathrm{the}\;\mathrm{AC}}\times 100$$

## 3. Results and Discussion

### 3.1. Thermogravimetric Analysis

**Figure 1:**
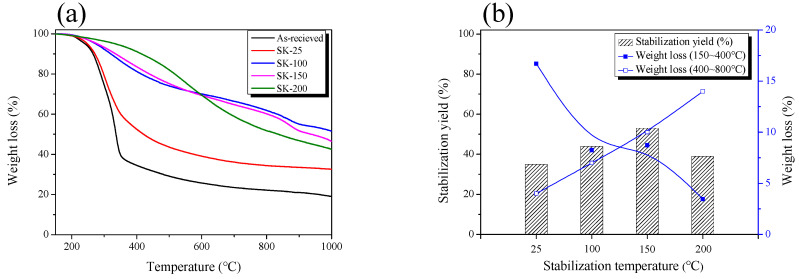
Thermal properties of stabilized kenaf: (**a**) Thermogravimetric analysis (TGA) curves; (**b**) weight loss.

Thermogravimetric analysis (TGA) is a useful analytical method to observe the carbonization behavior of the carbon material precursor. It is well known that lignocellulosic biomass such as kenaf at room temperature undergoes dehydration, degradation, and condensation (crosslinking) by phosphoric acid [26]. The TGA was used to observe the effect of the phosphoric acid stabilization temperature on the kenaf. Figure 1a shows TGA curves of stabilized kenaf (SK) with various phosphoric acid stabilization temperatures. After stabilization at various temperatures, kenaf was washed with distilled water until the pH of the filtrate reached pH 7. The TGA analysis of all washed kenaf with various stabilization temperatures was examined under a pure nitrogen atmosphere.

As the stabilization temperature increased, the stabilization yield of kenaf increased up to 423 K and then decreased. The reaction rate of dehydration and degradation increases as the stabilization temperature of phosphoric acid increases. Phosphorus compounds can form ester linkages with –OH groups on stabilization temperatures of cellulose below 473 K, helping to crosslink the polymer chains [27]. In other words, the increase in the stabilization yield of kenaf is considered as a result of the crosslinking by the phosphorus compound.

The TGA curve of as-received kenaf can be classified into step 1 (pyrolysis, 423–673 K) and step 2 (consolidation of the char structure, 673–1073 K). Cellulose, hemicellulose, and lignin constituting the kenaf are thermally decomposed in step 1 to form C ring (amorphous carbon). And in step 2, the amorphous carbon condenses and grows into larger sheets and stacks, and phosphate linkages are thermally decomposed [26,27,28,29]. In this process, the loss of volatile components that are not strongly bonded to the solid phase increases and the carbonization yield reduces.

SK-25 had the lowest stabilization and carbonization yield among the as-received and stabilized kenaf, and the only thermal decomposition was observed in step 1. In other words, it is judged that the dehydration, decomposition, and condensation of kenaf by phosphoric acid at 298 K were not completely achieved due to the slow reaction rate. The TGA curves of SK-100 and SK-150 showed no thermal decomposition in step 1, and a higher carbonization yield than the as-received kenaf. These results indicate that as the stabilization temperature increased, the kenaf was dehydrated and condensed by phosphoric acid and crosslinked at the same time. The lowest stabilization yield of kenaf stabilized at 473 K was observed, which is judged to be the effect of the increased decomposition and dehydration reaction rates due to the high stabilization temperature [27].

Figure 1b observed the weight loss (step 1 (423–673 K) and step 2 (673–1073 K)) of stabilized kenaf at various stabilization temperatures. In step 1, as-received kenaf was observed as the largest weight loss, and as the stabilization temperature increased, the weight loss of the cross-linked kenaf gradually decreased. As the stabilization temperature increased, it was considered that the weight loss of kenaf decrease due to an increase in decomposition or dehydration by phosphoric acid in the stabilization process. On the other hand, as the stabilization temperature increased in step 2, the weight loss of kenaf gradually increased. In step 2, amorphous are stacked up and expanded to form a turbostratic structure, and released as H_2_, CH_4_, CO, CO_2_ by dehydration and decarboxylation reactions, resulting in weight loss [29]. Stabilized kenaf by phosphoric acid contained a large amount of phosphate linkages in the structure, and phosphate linkages were observed to be thermally decomposed at 723 K or higher [26]. As the stabilization temperature increases, phosphate linkages in the stabilized kenaf significantly increased, resulting in the increased cross-linking density. Afterwards, phosphate linkages were decomposed in step 2, increasing the weight loss.

### 3.2. X-ray Diffraction Analysis

**Figure 2:**
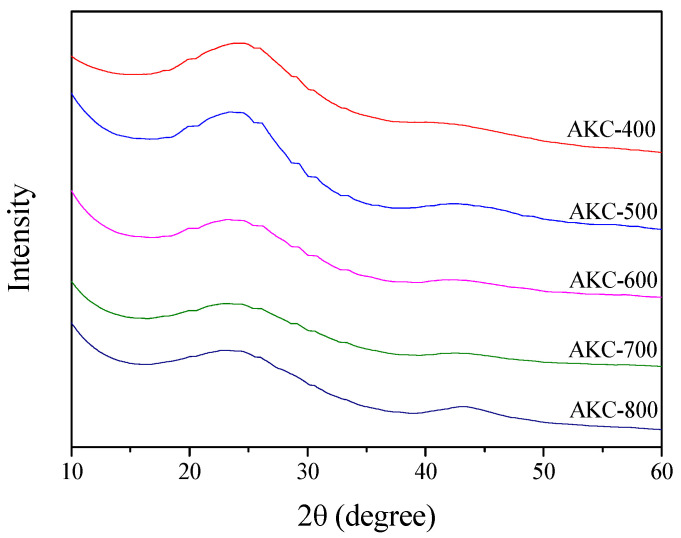
X-ray diffraction patterns of kenaf-derived activated carbons as a function of various phosphoric acid activation temperature.

**Figure 3:**
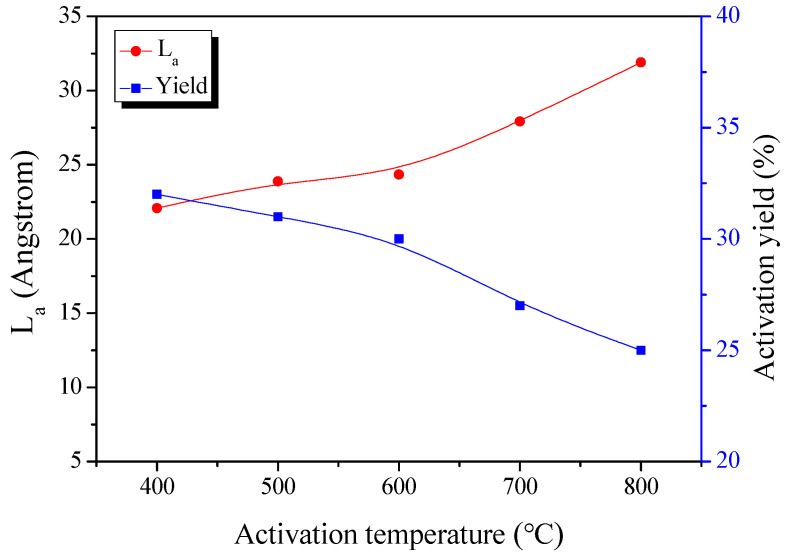
Structural characteristics of kenaf-derived activated carbons as a function of various phosphoric acid activation temperature.

XRD is a useful analytical method that can analyze the crystal structure of activated carbon during activation. Figure 2 and Figure 3 shows the X-ray diffraction curve of AKC and the crystal size (L_a_) calculated therefrom. As the activation temperature increased to 673–1073 K, L_a_ and activation yield were observed to be 22.1–31.9 Å and 32–25%, respectively. In addition, L_a_ was observed to have a very close correlation with the activation yield. The crystal structure of AKC can be classified into 673–873 K and 873–1073 K depending on the change in L_a_. As the activation temperature increased, L_a_ increased to a small width to 873 K and then increased to a large width.

The activation mechanism of lignocellulose by phosphoric acid is known as the reaction of decomposition of phosphate linkages, consolidation of the char structure, oxidation of crystals, etc. [26]. First, lignocellulose reacts with phosphoric acid to form a polyphosphate ester, whereby a crosslinking occurs. As a result, kenaf stabilized by phosphoric acid contains a large amount of phosphate linkages in the structure. At an activation temperature of 723 K or higher, phosphate linkages in the crystal structure are thermally decomposed, thereby expanding the pore structure and developing mesopores [26]. Second, lignocellulose is decomposed and dehydrated during the stabilization process to form amorphous [26]. As the activation temperature increases, as amorphous changes into a turbostratic structure, micropores are formed, and elements such as H and O are released into gas, resulting in weight loss. Third, oxidation primarily occurs in amorphous or smaller crystallite than crystalline, so that L_a_ is observed as a relative increase [30]. It was considered that L_a_ grows under the influence of crystal growth and crystal oxidation with increasing activation temperature.

In Figure 3, the activation yield of kenaf was observed as a nonlinear curve with decreasing slope at 873 K. However, in the TGA analysis of Figure 1, the carbonization yield of stabilized kenaf decreased linearly due to turbo layer deposition and decomposition of phosphate linkages. According to Elmouwahidi’s research, the amount of CO and CO_2_ evaporated due to phosphoric acid activation of biomass rapidly increase at above 873 K [31]. In other words, it is judged that the activation yield decreases due to the oxidation of crystal grains by oxygen formed in the process of decomposition of phosphate linkages above an activation temperature of 873 K. Therefore, the increase in L_a_ due to the increase in the activation temperature is judged to be influenced by turbostratic stacking and amorphous oxidation. Especially at 873 K or more, the oxidation reaction of grains increases, which greatly increases L_a_.

### 3.3. Adsorption Isotherm and Textural Properties

**Figure 4:**
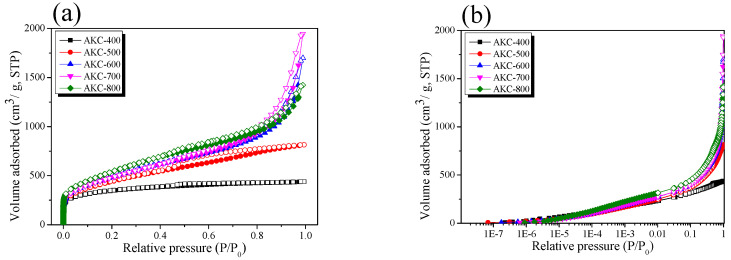
N_2_/77 K isotherm adsorption-desorption curves of kenaf-derived activated carbons as a function of various phosphoric acid activation temperature; (**a**) normal and (**b**) logarithmic.

The N_2_/77 K isothermal adsorption-desorption curve is the most useful analytical method for analyzing the textural properties of activated carbon. Figure 4 displays the isothermal adsorption-desorption curves of kenaf-derived activated carbons according to various phosphoric acid activation temperatures. The isothermal adsorption/desorption curve of AKC changed from Type I to Type II of the IUPAC classification as the activation temperature increased. The isothermal adsorption curve of AKC-400 falls under Type I of the IUPAC classification [32]. The adsorption curve of AKC-400 was observed only at a relative pressure (P/P_0_) of 0.05 or less. These results are shown by single-layer adsorption caused by strong interaction between the pore wall of the activated carbon and the adsorbent (N_2_), and mean activated carbon with micropores developed. The isothermal adsorption curves of AKC-500 to AKC-800 were observed for both adsorption at a relative pressure (P/P_0_) of 0.05 or less, and adsorption at a relative pressure (P/P_0_) of 0.05 or more. These results are not only due to single-layer adsorption, but also multi-layer adsorption of pore walls and adsorbents, and both micropores and mesopores are mainly observed in the developed activated carbon.

In Figure 4b, all AKC curves were observed with almost similar adsorption behavior up to the relative pressure (P/P_0_) of 0.01. Therefore, all AKCs have similar microporous structures regardless of the activation temperature. It is known that the phosphoric acid activation mechanism causes activation of the amorphous polymers (hemicellulose and lignin) produces mostly micropores at a temperature of 723 K or lower, while activation of crystalline cellulose produces a mixture of pore sizes at a temperature of 723 K or higher [26]. In other words, it is determined that micropores are generated by dehydration of amorphous polymers up to AKC-400, and mesopores are generated in AKC-500 to ACK-800 by decomposition of phosphate linkages.

Isothermal adsorption curve hysteresis of AKC was influenced by activation temperature. The hysteresis of AKC-400 to AKC-500 and AKC-600 to AKC-800 were observed as IUPAC standards Type H4 and Type H3, respectively [32]. Therefore, the pore shapes of AKC-400 to AKC-500 and AKC-600 to AKC-800 are considered to have narrow slit-like pores and slit-like pores, respectively. AKC forms a pore in a narrow slit-like pore shape at the beginning of activation, but as the activation temperature increases, it becomes a slit-like pore shape. The phosphoric acid activation mechanism is achieved by various chemical reactions such as decomposition of phosphate linkages, turbostratic stacking, and crystal oxidation, and is known to have a different reaction rate depending on the activation temperature. Therefore, AKC prepared at various activation temperatures is thought to form different pore structures despite having similar activation yields.

Table 1 exhibited the textural properties of AKC according to the activation temperature. The specific surface area and total pore volume of AKC were observed to be 1260 to 1810 m^2^/g and 0.68 to 2.77 cm^3^/g, respectively. In an activation temperature of 273–673 K, the specific surface area rose sharply from 1260 m^2^/g due to the development of micropores. It is judged that ACK-400 forms pore by oxidation of amorphous by phosphoric acid, thereby forming micropore-rich pore structures. In an activation temperature of 773–873 K, the micropore and mesopore volume increased from 0.56–0.61 cm^3^/g and 0.70–1.82 cm^3^/g, respectively. In particular, the mesopore volume increased larger than the micropore volume, and the proportion of mesopores increased despite the increase of the micropores. As mentioned earlier, AKC prepared by phosphoric acid activation contains a large amount of phosphate linkages in the structure. It is known that the decomposition of phosphate linkages at an activation temperature of 723 K or more expands pore structure and develops mesopores [26]. As the activation temperature increased, the oxidation of crystals and the decomposition of phosphate linkages increased, and thus it was considered that the volume of micropores and mesopores increased. The AKC-700 did not change micropores, but the volume of the mesopores increased significantly. Figure 1 showed that the activation yield is greatly reduced by increasing the crystal oxidation reaction at above 873 K or more. AKC-700 is thought to have a large increase in the volume of mesopores due to the collapse of micropores due to the amorphous oxidation due to the increase in the oxidation rate. In the AKC-800, the micropore volume increased from 0.60 to 0.67 cm^3^/g, while the mesopore volume decreased from 2.17 to 1.44 cm^3^/g. Unlike other AKCs, it is considered that the AKC-800 has formed a turbostratic structure due to its high activation temperature. It is also known that crystal shrinkage occurs during the formation of a turbostratic structure. In other words, the pore structure expanded by decomposition of phosphate linkages contracted again while forming a turbostratic structure, thereby reducing the mesopores. In addition, it is considered that the micropore volume increased due to high amorphous oxidation.

**Figure 5:**
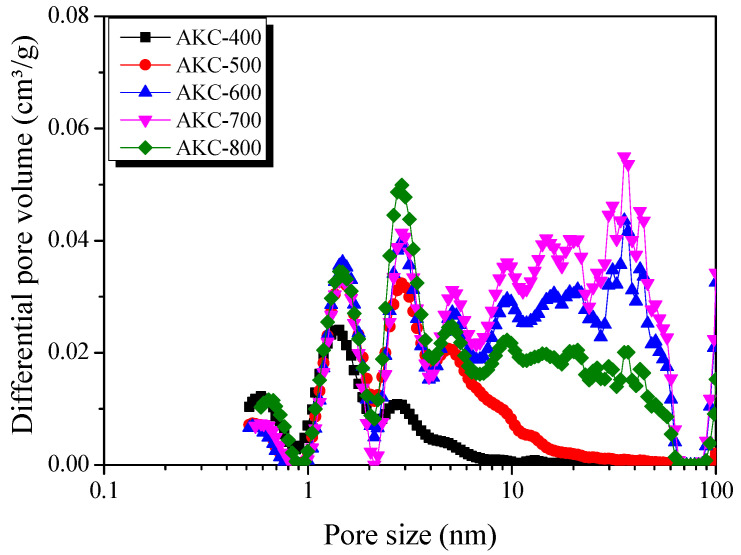
Pore size distribution of kenaf-derived activated carbons as a function of various phosphoric acid activation temperature.

NLDFT is the most powerful analysis method for observing the pore size distribution (PSD) of activated carbon based on thermodynamics. Figure 5 exhibited the PSD curve of AKC according to the activation temperature. PSD exhibited a similar trend to textural properties. AKC-400 has a PSD curve mainly developed with micropores and sub-mesopores of 3 nm or less. AKC-400 has the highest pore volume in pores with a diameter of less than 1 nm among kenaf derived activated carbon by amorphous oxidation.

At the activation temperature of 773 K, the pore volume in pores with diameters of 1 nm or less was decreased, but the pore volume in the sub-mesopore with diameters of 2–7 nm was significantly increased. At an activation temperature above 723 K, phosphate linkages in the structure decomposed, thereby causing amorphous condensed by phosphoric acid to separate from the crystal grains. It is considered that the mesopores were developed by oxidation of separated amorphous.

As the activation temperature increased, PSD of AKC-600 and AKC-700 showed an increase in pore volume in pore with diameters of 10–50 nm. It is believed that the increase in the volume of the mesopores was caused by the crystal oxidation reaction and the decomposition of phosphate linkages due to the increase in the activation temperature.

In AKC-800, the pore volume of the sub-mesopore with a pore diameter of 2.0–7.0 nm increased and the pore volume with a pore diameter of 10–50 nm decreased. Above 923 K, the crystal structure of the carbon material shrinks as the amorphous material changes to a turbostratic structure. Phosphoric acid activation causes mesopore to develop due to the expansion of pore structure by the decomposition of phosphate linkages. In other words, it is assumed that the mesopore volume decreased as the expanded pore structure by phosphatic acid contracted again as the AKC-800 formed a turbostratic structure.

### 3.4. Butane Working Capacity

Butane working capacity (BWC) is the most well-known analytical method for analyzing activated carbon canister performance. BWC is analyzed through adsorption and desorption behavior of activated carbon on n-butane. As shown in Equations (2)–(4), butane activity and butane retentivity are calculated by the adsorption ratio and the residual ratio after desorption, respectively.

**Figure 6:**
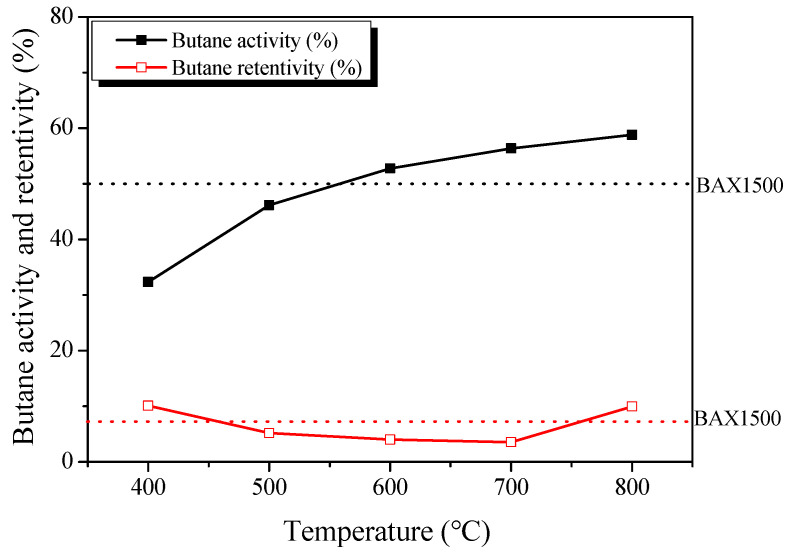
Butane working capacity of kenaf-derived activated carbons as a function of various phosphoric acid activation temperature.

Figure 6 exhibited the BWC of AKC. As the activation temperature increased, butane activity increased from 32.34 to 58.81%. On the other hand, the butane retentivity decreased to 10.12–3.55% from 673 to 973 K as the activation temperature increased, and then increased to 9.96% at 1073 K. Through many studies, it is known that the adsorption and desorption capacity of activated carbon is determined by the volume of micropores and mesopores, respectively [21]. In Table 1, the micropore volume of AKC increased to 0.47–0.67 cm^3^/g as the activation temperature increased. On the other hand, the volume of the mesopores increased from 0.21 to 2.17 cm^3^/g up to 973 K as the activation temperature increased, and then decreased to 1.44 cm^3^/g at 1073 K. In other words, it was confirmed that the butane activity and retentivity depend on the micropore and mesopore volume, respectively.

In addition, BWC, which stands for butane adsorption and desorption behavior of activated carbon, increased to 22.2–52.8% up to 973 K as the activation temperature increased, and then decreased to 48.8% at 1073 K. BWC is an index reflecting both butane activity and retentivity. Therefore, AKC-700 is considered to have the best BWC performance because it has excellent butane activity and the lowest butane retentivity. On the other hand, although AKC-800 has the most excellent butane activity, it has high butane retentivity compared to AKC-700, so it is considered to have BWC performance similar to AKC-600. ACK-700 was found to have about 5% better BWC because it has better butane activity and retentivity than BAX1500, activated carbon for commercial canisters [21].

**Figure 7:**
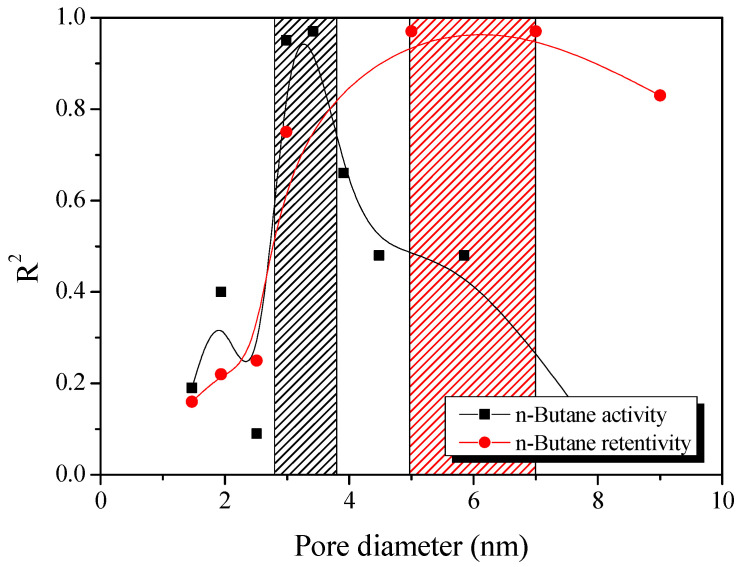
Correlations between the butane activity and retentivity of kenaf-derived activated carbons with pore volume.

Figure 7 exhibits the result of plotting the pore volume according to pore diameter in 0.5 nm units using the NLDFT method and then plotting the coefficient of determination with butane activity and retentivity. It is considered that the butane activity and retentivity of the AKC are determined by pores with diameters from 2.8–3.8 nm and 5.0–7.0 nm, respectively. Therefore, the butane adsorption and desorption capacity of AKC has a very close correlation with the volume of pore having 2.8–3.8 nm and 5.0–7.0 nm, respectively. This result is similar with those in previous studies [21,33] showing that sub-mesopores (1.5–3.0) and mesopore (3 nm or more) are essential for providing the butane adsorption and desorption capacity, respectively.

## 4. Conclusions

In this study, activated carbon with a high mesopore volume fraction was prepared using kenaf. The effect of various stabilization and activation temperatures on the pore structure of activated carbon was investigated. From the results, the micropore volume and mesopore volume of the AKC was determined to be 0.47–0.67 m^2^/g and 0.21–2.17 cm^3^/g, respectively. The textural properties (specific surface area, and mesopore volume fraction) could be arbitrarily controlled depending on the phosphoric acid activation conditions. The BWC of AKC has a close relationship with the sub-mesopore volume. It is also inferred that the mesopore volume fraction in the range of 2.8–3.8 nm and 5.0-7.0 nm primarily controls n-butane adsorption and desorption, respectively. Under the enhanced criteria pollutant exhaust regulations ever for vehicles, an effective adsorbent is required for an evaporative emission control system with an excellent butane working capacity (high butane activity and low retentivity). The AKC was confirmed to have superior BWC performance compared to commercial activated carbon (BAX1500) due to its excellent specific surface area and mesopore volume fraction.

## Figures and Tables

**Table 1:** Textural properties of kenaf-derived activated carbons as a function of various phosphoric acid activation temperature.

Sample	Activation Conditions	S_BET_ (m^2^/g)	V_Total_^1^ (cm^3^/g)	V_Micro_^2^ (cm^3^/g)	V_Meso_^3^ (cm^3^/g)	Activation Yield ^4^ (%)
AKC-400	673 K, 1 h	1260	0.68	0.44	0.24	32
AKC-500	773 K, 1 h	1510	1.26	0.44	0.72	31
AKC-600	873 K, 1 h	1650	2.43	0.43	2.00	30
AKC-700	973 K, 1 h	1620	2.77	0.39	2.38	27
AKC-800	1073 K, 1 h	1810	2.11	0.48	1.63	25

^1^ V_Total_: Total pore volume; The amount adsorbed P/P_0_ = 0.99. ^2^ V_Micro_: Micropore volume; pore volume of micropores (2 nm) was calculated by cumulative pore volume using NLDFT model. ^3^ V_Meso_: Mesopore volume; V_Total_-V_Micro_. ^4^ Activation yield: $\frac{\mathrm{Weight}\;\mathrm{of}\;\mathrm{activated}\;\mathrm{sample}}{\mathrm{Weight}\;\mathrm{of}\;\mathrm{carbonized}\;\mathrm{sample}\;\mathrm{input}}\times 100$.

## Data Availability

The data presented in this study are available on request from the corresponding author.
